# SPRY4 promotes adipogenic differentiation of human mesenchymal stem cells through the MEK–ERK1/2 signaling pathway

**DOI:** 10.1080/21623945.2022.2123097

**Published:** 2022-09-15

**Authors:** Na Li, Yunfei Chen, Haiyan Wang, Jing Li, Robert Chunhua Zhao

**Affiliations:** a Institute of Basic Medical Sciences Chinese Academy of Medical Sciences, School of Basic Medicine Peking Union Medical College, Center of Excellence in Tissue Engineering, Chinese Academy of Medical Sciences; Beijing Key Laboratory of New Drug Development and Clinical Trial of Stem Cell Therapy (BZ0381), P.R. China; b College of Basic Medicine, Shaanxi University of Chinese Medicine, Xianyang, P.R. China; c Department of Cell Biology, School of Life Sciences, Shanghai University, Shanghai, P.R. China

**Keywords:** SPRY4, hAMSCs, adipogenic differentiation, ERK1/2, obesity

## Abstract

Obesity is a chronic metabolic disorder characterized by the accumulation of excess fat in the body. Preventing and controlling obesity by inhibiting the adipogenic differentiation of mesenchymal stem cells (MSCs) and thereby avoiding the increase of white adipose tissue is safe and effective. Recent studies have demonstrated that Sprouty proteins (SPRYs) are involved in cell differentiation and related diseases. However, the role and mechanism of SPRY4 in MSC adipogenic differentiation remain to be explored. Here, we found that SPRY4 positively correlates with the adipogenic differentiation of human adipose-derived MSCs (hAMSCs). Via gain- and loss-of-function experiments, we demonstrated that SPRY4 promotes hAMSC adipogenesis both in vitro and in vivo. Mechanistically, SPRY4 functioned by activating the MEK–ERK1/2 pathway. Our findings provide new insights into a critical role for SPRY4 as a regulator of adipogenic differentiation, which may illuminate the underlying mechanisms of obesity and suggest the potential of SPRY4 as a novel treatment option.

## Introduction

Obesity has become a major public health challenge, and the number of obese people has increased dramatically over the past few decades [1–3]. In essence, obesity is caused by an increase in the mass of white adipose tissue (WAT), including an abnormal increase in the number and volume of adipocytes [4]. Adipogenesis is a process in which preadipocytes differentiate into mature adipocytes, leading to WAT expansion [5]. Therefore, adipogenesis can be used as a handle to control WAT accumulation, which offers an effective strategy for treating and preventing obesity [6]. MSCs are multipotent cells that can differentiate into chondroblasts, osteoblasts and adipocytes [7]. As they are a major source of adipocytes, understanding the specific mechanism of MSC adipogenesis is of great importance. Multiple signalling pathways, in concert with various transcription factors, regulate MSC adipogenic differentiation precisely [8–10]. However, the important functional regulators involved in MSC adipogenesis remains to be well defined.

SPRYs are highly conserved protein families and were originally identified as novel FGFR signalling antagonists during tracheal development in *Drosophila*. They were later proven to be inhibitors of receptor tyrosine kinase (RTK) signalling [11]. SPRYs have varied biological significance, including the regulation of cell proliferation, differentiation, cytoplasmic calcium concentration and cell migration [11,12]. Recently, it was reported that SPRYs play a critical role in adipogenesis. *SPRY1* was upregulated after weight loss interventions, and SPRY1 depletion in human adipogenic stromal cells by RNA interference reduced the expression of the early adipogenic transcription factor CCAAT/enhancer binding protein-β (C/EBPβ), with the abrogation of adipogenesis [13]. CRISPR/Cas9-mediated SPRY1 knockout in human adipogenic stromal cells also leads to impaired adipogenesis [14]. However, conflicting results have been observed. Fat-specific deletion of SPRY1 in mouse model induced total body fat accumulation, while SPRY1 gain of function led to reduced total body fat and hypertrophy of abdominal fat cells [15]. In vitro studies have shown SPRY1 suppresses adipocyte differentiation in the bone marrow MSCs [15]. Gonadal WAT expanded in SPRY1 knockout mice, and loss of SPRY1 promotes adipocyte differentiation [16]. Downregulaion of SPRY4 had different effects on MSC fate determination. SPRY4 suppression enhanced MSC osteogenic differentiation and bone formation, while inhibited chondrogenic differentiation and had no effect on the adipogenic differentiation of MSC in vitro [17]. However, a recent study found that SPRY4 inhibits osteogenic differentiation and induced adipogenic differentiation of mouse marrow stromal progenitor cells (including marrow-derived MSCs) in vitro and in vivo. Mechanistic studies suggested SPRY4 plays a key role in stem cell lineage commitments through inactivating ERK1/2-Wnt/β-catenin regulatory loop [18].

Previously, we found that SPRY4 regulates the osteogenic differentiation of bone marrow-derived MSCs and that it was implicated in the pathogenesis of adolescent idiopathic scoliosis [19]. The present study was conducted to explore whether SPRY4 affects the adipogenic differentiation of hAMSCs.

## Material and methods

### Antibodies and key reagents

Antibodies against Peroxisome proliferator-activated receptor gamma (PPARγ), CCAAT/enhancer binding protein-α (C/EBPα), fatty acid binding protein 4 (FABP4), and lipoprotein lipase (LPL) were from Abcam (Cambridge, MA, USA). Antibodies against SPRY4 and GAPDH were from Proteintech (Wuhan, China). Antibodies against phosphorylated (p)-ERK1/2, ERK1/2 and Perilipin 1 were from Cell Signalling Technology (Danvers, MA, USA). U0126 was from Selleck Chemicals (Houston, TX, USA).

### Isolation, culture and adipogenic differentiation of hAMSCs

hAMSCs were isolated and expanded as previously described [20]. Briefly, human adipose tissues were collected from donors undergoing liposuction surgery. The cells were isolated, and then cultured in a humidified incubator at 37°C with 5% CO2. Passage 3 hAMSCs were used in the following experiments. All experiments followed the procedures approved by the Ethics Committee of the Chinese Academy of Medical Sciences and Peking Union Medical College (NO.2019002) .

For adipogenic differentiation, hAMSCs were seeded at a density of 3 × 10^5^ cells in 6-well plates with basic growth medium. After the cells had reached 90% confluence, the culture medium was replaced with adipogenic medium composed of DMEM(Gibco) supplemented with 10% foetal bovine serum (Gibco), 1 μM dexamethasone (Sigma-Aldrich), 0.5 mM isobutylmethylxanthine (Sigma-Aldrich), and 1 mM L-ascorbic acid (Sigma-Aldrich). The medium was changed every other day during adipogenic differentiation.

### Oil red O staining and quantification

As previously described [21], oil red O staining was performed after the 10-day adipogenic differentiation. Briefly, cells were washed twice with phosphate-buffered saline (PBS) and fixed in 4% paraformaldehyde (500 μL/well) for 10 min. Then, the cells were stained with filtered oil red O solution (stock solution: 1 mg/mL in isopropanol; working solution: 60% oil red O stock solution mixed with 40% double-distilled water) for 30 min at 37°C. After staining, the cells were washed with double-distilled water to remove the unbound dye and were subsequently imaged. For quantification of oil red O staining, 100–200 μL isopropanol was added to each well, and the OD value was measured at a wavelength of 510 nm with an ELISA microplate reader.

### Small interfering RNA (siRNA) transfection and virus infection

For transient knockdown of SPRY4, hAMSCs were transfected with siRNAs via Lipofectamine 2000 (Life Technology) according to the manufacturer’s recommendations. The SPRY4 siRNAs and negative control (NC) synthesized by GenePharma (Shanghai, China) are listed in Supplementary Table S1. For stable knockdown in the animal experiments in vivo, the same siRNA sequences were inserted into LV3-pGLV-H1-GFP+Puro lentiviral vector, then lentivirus short hairpin RNAs (shRNAs) expression vector was constructed and packaged. For overexpression, the coding sequence of SPRY4 was cloned into a lentivirus vector (pEZ-Lv225), which was packaged by GeneCopoeia (Guangzhou, China). To obtain stable cell lines, hAMSCs were infected with lentivirus (multiplicity of infection [MOI] = 10) for 24 h, followed by treatment with 1 μg/mL puromycin (Sigma-Aldrich).

### Heterotopic lipid formation in vivo

hAMSCs with SPRY4 overexpression, SPRY4 knockdown, or the corresponding controls (generated by lentivirus infection) were incubated in adipogenic medium for 4 days. Then, 2 × 10^6^ cells were mixed with 150 µL Matrigel (BD Biosciences), and then injected subcutaneously into two symmetrical sites on the upper dorsal surface of 6-week-old BALB/c nu/nu female mice (n = 5 per group). After 8 weeks, the cells/Matrigel implants were obtained and fixed in 4% paraformaldehyde. All animal experiments were performed with the approval of the Ethics Committee of the Chinese Academy of Medical Sciences and Peking Union Medical College.

### HE staining and Immunofluorescence

Heterotopic lipid formation was observed using haematoxylin eosin (HE) staining, and immunofluorescence for Perilipin 1 was used to observe the efficiency of hAMSC adipogenic differentiation in vivo. Briefly, paraffin sections with a thickness of 3 μm were prepared according to standard protocols, and then dewaxed in xylene, rehydrated through decreasing concentrations of ethanol, and washed in PBS. For HE staining, the sections were stained with haematoxylin and eosin. After staining, sections were dehydrated through increasing concentrations of ethanol and xylene and mounted. For immunofluorescence, tissue antigens were retrieved by citric acid buffer (PH6.0) microwave antigen retrieval (Servicebio, Wuhan, China) at first. Then, the sections were blocked for 30 min at room temperature in 10% BSA (Servicebio), perilipin 1 was detected by incubation with anti- perilipin 1 antibody (Cell Signalling Technology) at 4°C overnight. Sections were rinsed three times in PBS and incubated with Goat anti-Rabbit fluorescent secondary antibody (Servicebio) for 1 h at room temperature the next day, and then cell nucleus were stained with DAPI (Servicebio) for 5 mins at room temperature. After rinsing three times in PBS, expression of perilipin 1 protein was observed under fluorescence microscope.

### RNA extraction and qRT-PCR

Total RNA was extracted from hAMSCs cultured in vitro using TRIzol (Invitrogen, Waltham, MA, USA) and then treated with DNase I (Ambion, Austin, TX, USA) at 37°C for 30 min. cDNA was synthesized using a reverse transcription kit (Takara, Otsu, Japan) according to the manufacturer’s instructions. qRT-PCR was conducted with Hieff™ qPCR SYBR® Green Master Mix (Yeasen, Shanghai, China) using QuantStudio 3 (Applied Biosystems, Foster City, CA, USA). The relative expression of each gene was evaluated using the comparative threshold cycle (2^−ΔΔCt^) method and normalized to the mRNA level of GAPDH. Supplementary Table S1 shows the primers used in this study.

### Western blotting

The cells were washed twice with cold PBS and then lysed with RIPA lysis buffer containing 1 mM protease inhibitor cocktail (Beyotime, Beijing, China). Total protein was collected and quantified with a BCA protein assay kit (Beyotime). Western blotting was performed as previously described [19]. Briefly, protein fractions (15–20 μg) were separated by SDS-PAGE gels and then transferred to 0.45 µm PVDF membranes (Millipore, Billerica, MA, USA). After blocking with 5% BSA for 1 h, the membranes were incubated with specific primary antibodies at 4°C overnight. Then, the membranes were incubated with HRP-conjugated secondary antibodies (Neobioscience, Shenzhen, China) for 1 h at room temperature after washing with TBST. The target protein bands were visualized by a chemiluminescence ECL reagent (Millipore).

## Statistical analysis

The experiments were repeated more than three times; all data were analysed by GraphPad Prism 7 (GraphPad Software Inc., La Jolla, CA, USA) and expressed as the mean ± standard deviation (SD). Statistical significance between two groups or among multiple groups was analysed using Student’s t-tests (two-sided) and one-way analysis of variance (ANOVA), respectively. Differences were considered statistically significant at * *p* < 0.05, ** *p* < 0.01 and *** *p* < 0.001.

## Results

### SPRY4 positively correlated with hAMSC adipogenic differentiation capacity

**Figure 1.**
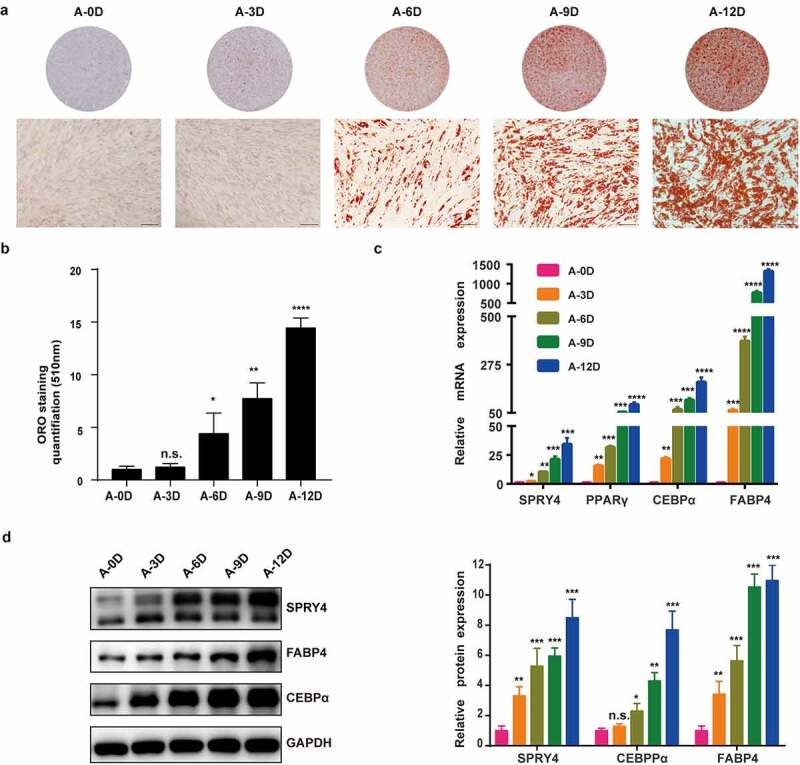
SPRY4 positively correlated with hAMSC adipogenic differentiation capacity.

We determined that all hAMSCs used in this study met the minimum standard for MSCs by the International Society for Cellular Therapy (Fig. S1). When passage 3 hAMSCs were 80–90% confluent, the culture medium was replaced by adipogenic medium for up to 12 days. The hAMSC adipogenic differentiation capacity was detected with oil red O staining, qRT-PCR and western blotting. The oil red O staining-positive rate increased gradually from 0 to 12 days (Figure 1a), and quantitative analysis confirmed this result (Figure 1b). qRT-PCR and western blotting also showed that the adipogenic transcription factors and marker genes PPARγ, C/EBPα and FABP4 were upregulated during adipogenic differentiation within 12 days (Figure 1c, d). Consistently, SPRY4 mRNA and protein levels were increased during adipogenic differentiation from day 0 to day 12 (Figure 1c, d). These data indicate that SPRY4 is positively associated with hAMSC adipogenic differentiation.

(a) Oil red O staining gradually increased from 0 to 12 days. (b) Quantification of Oil red O staining for evaluating the efficiency of hAMSC adipogenesis. (c) qRT-PCR detected the mRNA levels of SPRY4, PPARG, CEBPA and FABP4 mRNA levels during adipogenic differentiation (0 d, 3 d, 6 d, 9 d and12 d). (d) Western blotting showed the protein levels of SPRY4, C/EBPα and FABP4 during adipogenic differentiation (0 d, 3 d, 6 d, 9 d and 12 d), and quantitative analysis of the intensity of protein expression in the indicated groups. GAPDH was used as the control for normalization in the and western blotting. All data are the mean ± SD (n = 3 independent experiments with three biological repetitive tests, scale bar = 100 μm).

### Downregulation of SPRY4 impaired hAMSC adipogenic differentiation in vitro

**Figure 2.**
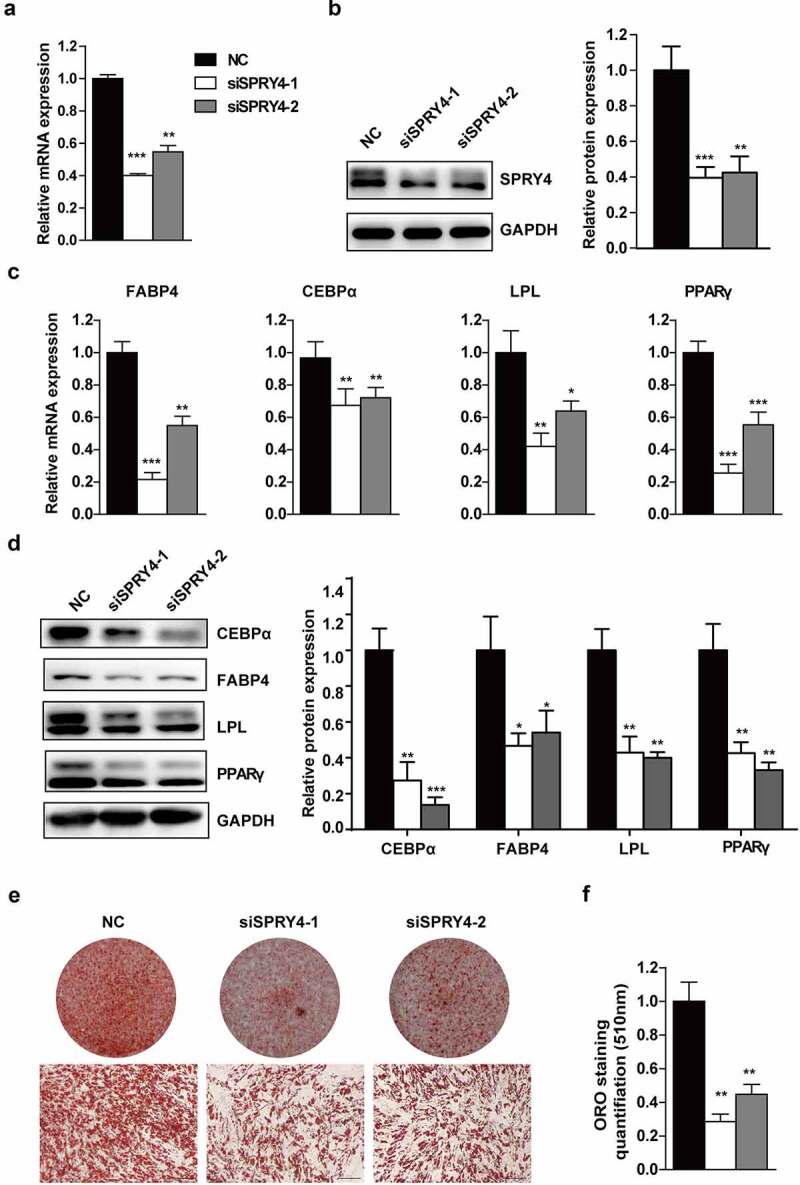
Downregulation of SPRY4 impaired hAMSC adipogenic differentiation in vitro.

To investigate the role of SPRY4 in hAMSC adipogenic differentiation, we silenced SPRY4 using two independent siRNAs. Compared to the NC, SPRY4 mRNA and protein levels were greatly downregulated in the knockdown groups (Figure 2a, b). Subsequently, the transfected hAMSCs were induced to differentiate into the adipogenic lineage. The mRNA levels of *PPARG, CEBPA, FABP4* and *LPL* were significantly downregulated with SPRY4 knockdown (Figure 2c). Meanwhile, expression of the adipogenic markers at protein level was attenuated (Figure 2d). Consistently, oil red O staining-positive cells and quantification (Figure 2e, f) further indicated that SPRY4 knockdown resulted in adipogenesis delay. These results prove that SPRY4 knockdown impairs hAMSC adipogenic differentiation in vitro.

SPRY4 was silenced in hAMSCs using two independent siRNAs (siSPRY4-1 and siSPRY4-2). Knockdown efficiency was verified by qRT-PCR at mRNA level (a) and western blotting at protein level (b). (c) qRT-PCR detected PPARG, CEBPA, FABP4 and LPL on day 6 of adipogenic differentiation. (d) Western blotting detected PPARγ, C/EBPα, FABP4and LPL on day 6 of adipogenic differentiation, and quantitative analysis of the intensity of protein expression in the indicated groups. (e, f) Oil red O staining and quantification were performed on day 10 of adipogenic differentiation to evaluate the efficiency of hAMSC adipogenesis after SPRY4 knockdown. GAPDH was used as the control for normalization in the qRT-PCR and western blotting. All data are the mean ± SD (n = 3 independent experiments with three biological repetitive tests, scale bar = 100 μm).

### Ectopic expression of SPRY4 promoted hAMSC adipogenic differentiation in vitro

**Figure 3.**
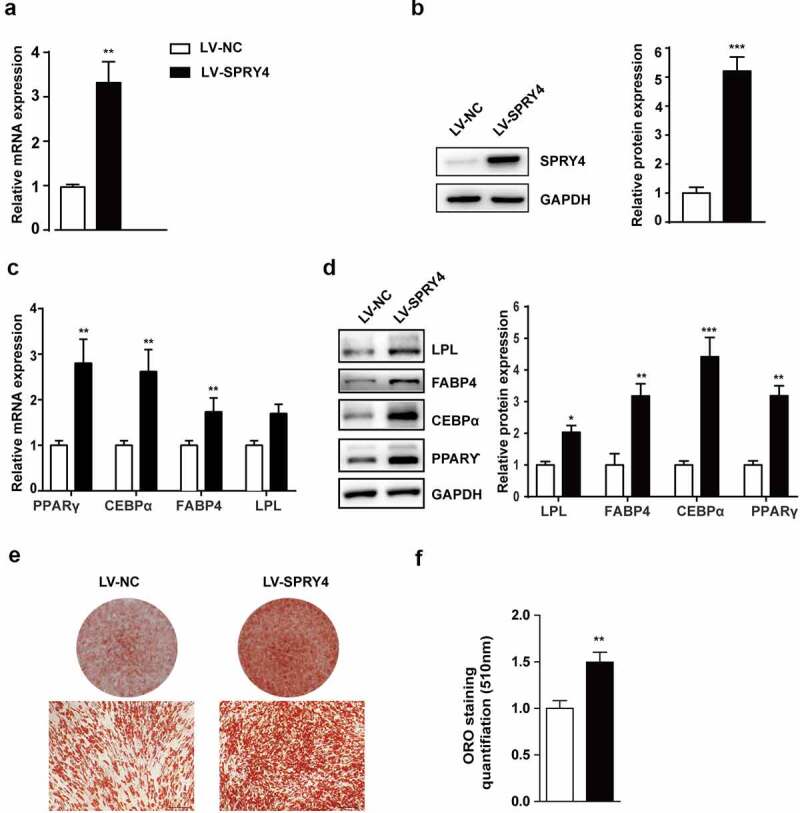
Ectopic expression of SPRY4 promoted hAMSC adipogenic differentiation in vitro.

To elucidate the effect of SPRY4 on adipogenic differentiation, we used lentivirus to overexpress SPRY4. qRT-PCR and western blotting showed that SPRY4 expression was upregulated effectively after the lenti-SPRY4 virus infection (Figure 3a, b). Next, the lentivirus-infected hAMSCs were induced towards the adipogenic lineage. Ectopic expression of SPRY4 significantly increased PPARγ, C/EBPα, FABP4 and LPL expression at both mRNA and protein level (Figure 3c, d). Furthermore, oil red O staining and quantification showed that more lipid droplets formed after lenti-SPRY4 infection, suggesting that there were more adipocytes and that hAMSC adipogenesis had been promoted (Figure 3e, f). Altogether, these data demonstrate that SPRY4 is a positive regulator of hAMSC adipogenic differentiation in vitro.

hAMSCs was infected with specific lentivirus to overexpress SPRY4. The efficiency of ectopic expression was verified by qRT-PCR at mRNA level (a) and western blotting at protein level (b). (c) qRT-PCR detected PPARG, CEBPA, FABP4 and LPL on day 6 of adipogenic differentiation. (d) Western blotting detected PPARγ, C/EBPα, FABP4 and LPL on day 6 of adipogenic differentiation, and quantitative analysis of the intensity of protein expression in the indicated groups. (e, f) Oil red O staining and quantification were performed on day 10 of adipogenic differentiation to evaluate the efficiency of hAMSC adipogenesis after ectopic expression of SPRY4. GAPDH was used as the control for normalization in the qRT-PCR and western blotting. All data are the mean ± SD (n = 3 independent experiments with three biological repetitive tests, scale bar = 100 μm).

### SPRY4 accelerated hAMSC heterotopic lipid formation in vivo

**Figure 4.**
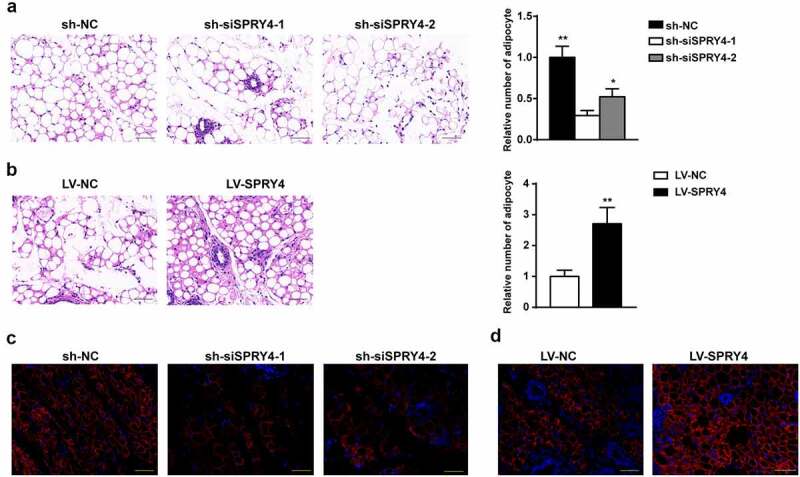
SPRY4 accelerated hAMSC heterotopic lipid formation in vivo.

To assess the potential effect of SPRY4 in vivo, we used a heterotopic lipid formation model in nude mice. hAMSCs were incubated in adipogenic medium for 4 days after treatment with sh-NC, sh-siSPRY4-1, sh-siSPRY4-2, LV-NC, or LV-SPRY4 lentivirus. After pre-induction, the same number of hAMSCs were mixed with Matrigel and then injected subcutaneously into the upper dorsal surface of the mice. Eight weeks later, the preadipocytes/Matrigel implants were harvested for HE staining and immunofluorescence for perilipin 1. Perilipin 1, a major lipid droplet-binding protein, is a specific marker of adipogenic differentiation due to its high expression in adipocytes [22]. HE staining revealed that SPRY4 depletion (sh-siSPRY4-1 and sh-siSPRY4-2 groups) clearly reduced the number of adipocytes as compared to the sh-NC group, while more adipocytes were observed in the LV-SPRY4 group than in the LV-NC group (Figure 4a, b). Immunofluorescence demonstrated that the SPRY4-silenced group had fewer Perilipin 1^+^ adipocytes than the control group, whereas more were detected in the SPRY4-overexpressing group (Figure 4c, d). Collectively, SPRY4 overexpression in the hAMSCs accelerated heterotopic lipid formation in vivo, while SPRY4 knockdown had the opposite effect.

hAMSCs were infected with lentivirus (sh-NC, sh-siSPRY4-1, sh-siSPRY4-2, LV-NC, or LV-SPRY4), and then incubated in adipogenic medium for 4 days before being transplanted subcutaneously in vivo, which was detected after 8 weeks. (a, b) Heterotopic lipid formation was detected by HE staining, and quantitative analysis of relative number of adipocytes. (c, d) Immunofluorescence detected perilipin 1-positive cell numbers for representing the efficiency of hAMSC ectopic fat formation (n = 6, scale bar = 50 μm).

### MEK–ERK1/2 pathway inhibition suppressed hAMSC adipogenic differentiation

SPRY4 belongs to the SPRY family, which can regulate the MEK–ERK1/2 pathway [23]. To clarify the molecular mechanism by which SPRY4 regulates adipogenic differentiation, we detected ERK1/2 activation with western blotting. Fig. S2 shows that the knockdown of SPRY4 dramatically decreased the phosphorylation level of ERK1/2. Conversely, SPRY4 overexpression increased them.

**Figure 5.**
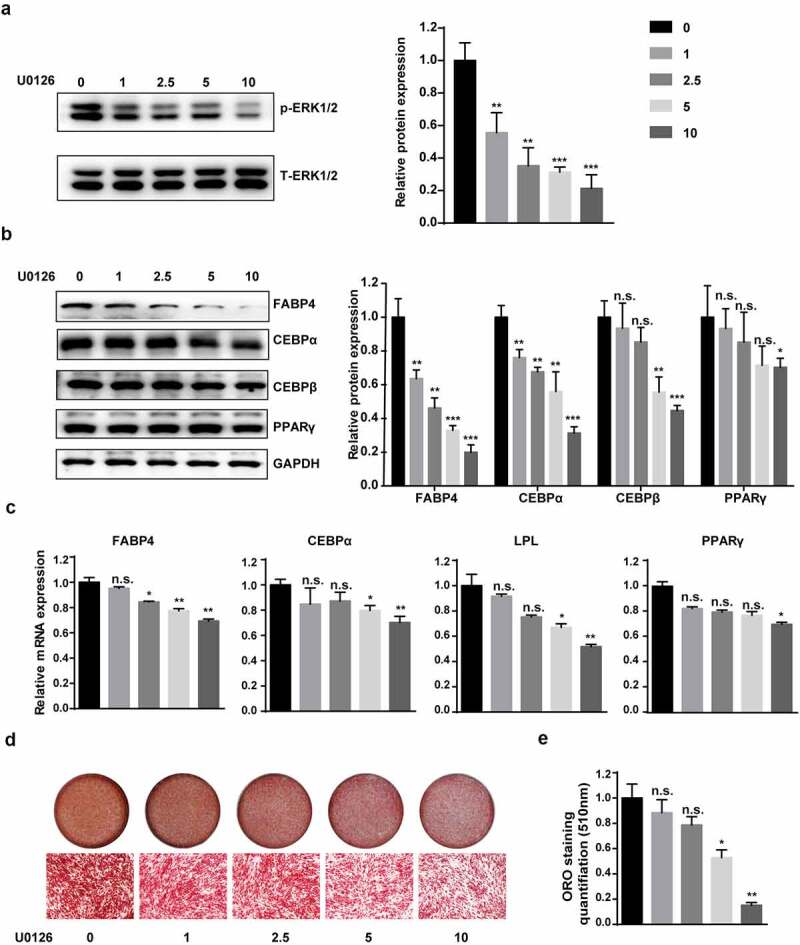
MEK–ERK1/2 pathway inhibition suppressed adipogenic differentiation.

However, the role of the MEK–ERK1/2 pathway in adipogenic differentiation and obesity has been the subject of contradictory reports [24–27]. To explore the correlation between the MEK–ERK1/2 signalling pathway and hAMSC adipogenic differentiation, we blocked the pathway using U0126, an ERK signalling pathway-specific inhibitor. We found that U0126 could decrease the phosphorylation level of ERK 1/2 in a concentration dependent manner (Figure 5a). During adipogenic differentiation, qRT-PCR and western blotting showed that U0126 notably downregulated C/EBPα, FABP4 and LPL expression in a concentration-dependent manner (Figure 5b, c). Consistent with these results, the gradually decreasing oil red O staining-positive cells and quantification confirmed that U0126 treatment resulted in impaired adipogenesis (Figure 5d, e). These results prove that inhibiting the MEK–ERK1/2 pathway suppresses hAMSC adipogenic differentiation in a concentration-dependent manner.

(a) U0126 (0, 1, 2.5, 5, 10 μM) was used to block the MEK–ERK1/2 pathway. (b) Western blotting detected PPARγ, C/EBPα, FABP4 and LPL, as well as total ERK1/2 (T-ERK) and p-ERK1/2 (p-ERK) on day 6 of adipogenic differentiation, and quantitative analysis of the intensity of protein expression in the indicated groups. (c) qRT-PCR detected PPARG, CEBPA, FABP4 and LPL on day 6 of adipogenic differentiation. (d, e) Oil red O staining and quantification were performed on day 10 of adipogenic differentiation to evaluate the efficiency of hAMSC adipogenesis after MEK–ERK1/2 pathway inhibition. GAPDH was used as the control for normalization in the qRT-PCR and western blotting analyses. All data are the mean ± SD (n = 3 independent experiments with three biological repetitive tests, scale bar = 100 μm).

### SPRY4 regulated hAMSC adipogenic differentiation via the MEK–ERK1/2 pathway

**Figure 6.**
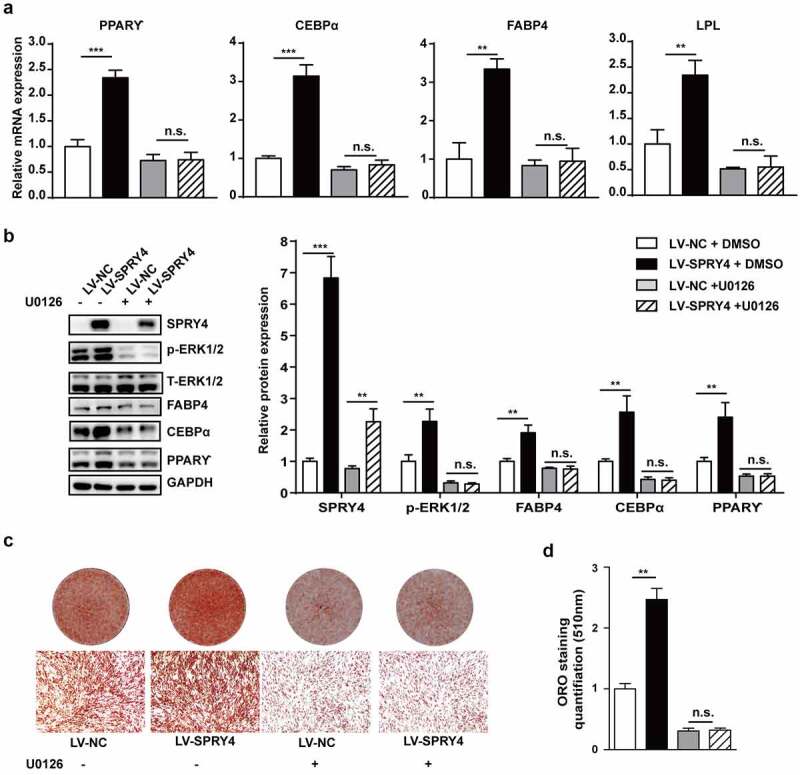
SPRY4 functioned by activating the MEK–ERK1/2 pathway.

Next, we attempted to determine whether the MEK–ERK1/2 pathway is responsible for the adipogenic-promoting effects of SPRY4. hAMSCs were infected with lentivirus to overexpress SPRY4, and then induced towards adipogenic lineage treating with 10 μM U0126. Figure 6b shows that U0126 almost blocked SPRY4 enhancement of the phosphorylation level of ERK1/2. Ectopic expression of SPRY4 increased PPARγ, C/EBPα, FABP4 and LPL expression, while U0126 abolished these results (Figure 6a, b). Oil red O staining and quantification also proved that blocking the MEK–ERK1/2 pathway abrogated the adipogenic-promoting abilities of SPRY4 (Figure 6c, d). Together, these data demonstrate that SPRY4 regulates hAMSC adipogenic differentiation via the MEK–ERK1/2 signalling pathway.

SPRY4 was overexpressed in hAMSCs, and then the cells were treated with DMSO or 10 μM U0126 during adipogenic induction. (a) qRT-PCR detected PPARG, CEBPA, FABP4 and LPL on day 6 of adipogenic differentiation. (b) Western blotting detected PPARγ, C/EBPα, FABP4 and LPL as well as SPRY4, T-ERK and p-ERK on day 6 of adipogenic differentiation, and quantitative analysis of the intensity of protein expression in the indicated groups. (c, d) Oil red O staining and quantification were performed on day 10 of adipogenic differentiation to evaluate the efficiency of hAMSC adipogenesis after different treatments. GAPDH was used as the control for normalization in the qRT-PCR and western blotting. All data are the mean ± SD (n = 3 independent experiments with three biological repetitive tests, scale bar = 100 μm).

## Discussion

Obesity is a major risk factor for a variety of diseases. The main feature of obesity is the massive increase in white adipose tissue, while the growth of adipose tissue involves an increase in adipocyte size and the formation of new adipocytes [28]. Therefore, inhibiting the fat-forming process is the fundamental approach for treating and preventing obesity. MSCs are original progenitor cells with multilineage differentiation potential and reproductive activity. MSCs can not only differentiate into osteoblasts, chondrocytes and adipocytes of the mesoderm, but can also differentiate across the germ layer under proper induction conditions [10]. As the common progenitor cells of adipocytes, MSCs are considered the promising target for obesity therapy. Many studies have examined the potential applications of MSCs for treating obesity, and some results are encouraging [29–31], which suggests that obesity can be avoided by controlling MSC adipogenic differentiation. However, MSC adipogenic differentiation is a highly complex process, and the specific molecular mechanisms remain ill-defined.

SPRYs were found in genetic screening for trachea [32] and eye [33] developmental regulators in *Drosophila*. In mammalians, four different *Spry* members (*Spry1*–*4*) have been identified. *Spry1, Spry2* and *Spry4* can be detected in various tissues and organs, whereas *Spry3* is only expressed in mouse testis and adult brain [34]. SPRYs have recently been implicated in MSC differentiation [15,18,35], making them promising therapeutic targets in related diseases. Herein, we show that SPRY4 positively correlates with the adipogenic differentiation capacity of hAMSCs. Further study revealed that knockdown of SPRY4 significantly inhibited the expression of the adipogenic transcription factors PPARγ and C/EBPα, strongly impairing the hAMSC adipogenesis process in vitro. Besides, we show that the ectopic expression of SPRY4 had the opposite effect. Consistently, a recent study has shown that SPRY4 expression was induced in bone marrow stromal cells (BMSCs) during adipogenic differentiation [18]. SPRY4 promoted BMSC adipogenic differentiation by inactivating ERK–Wnt–β-catenin signalling [18]. Mandl et al. found that SPRY1 is essential for the initiation of adipogenesis. Knockdown of SPRY1 by shRNAs inhibited adipogenesis by augmenting ERK signalling and preventing C/EBPβ expression [13]. In addition, they later reported that adipogenesis was abrogated in human adipose stem/progenitor cells with SPRY1 knockout by CRISPR/Cas9-mediated genome editing [14]. Therefore, we and others show that downregulating SPRYs prevents adipogenesis.

SPRYs are mainly considered a major class of negative feedback loop modulators of RTK-induced ERK1/2 activation [12,36]. However, we reveal an active effect of SPRY4 on the MEK–ERK1/2 pathway during hAMSC adipogenic differentiation. Knockdown of SPRY4 dramatically decreased ERK1/2 phosphorylation, while SPRY4 overexpression increased ERK1/2 phosphorylation. In fact, SPRYs do not always inhibit ERK1/2 activation [37–39]. SPRY1 and SPRY2 increased EGF-mediated ERK activation in endothelial HEK293, CHO and HeLa cells by binding c-Cbl protein and thereby weakening EGF receptor ubiquitination and degradation [40–43]. Therefore, the role of SPRYs in the RTK-mediated MAPK–ERK signalling pathway seems to be cell-specific and context-dependent. In addition, SPRYs have many binding partners, including different effectors of the MAPK signalling pathway. The intersection points where SPRYs interfere with the MAPK pathway and their interplay with other interacting molecules may partly explain the dual but opposite effects on MAPK activation [37,44].

Although the effect of the MEK–ERK1/2 signalling pathway on adipogenic differentiation has been addressed, the results obtained so far appear contradictory [24,25,45]. On the one hand, changes in molecular expression in the same cell type have different effects on the ERK signalling pathway and subsequently on adipogenic differentiation. Downregulating CTRP6 suppressed the differentiation of 3T3-L1 preadipocytes by inhibiting the ERK1/2 signalling pathway [46], while inhibition of the MEK–ERK1/2 signalling pathway by PD98059 had no inhibitory effect on 3T3-L1 preadipocyte adipogenesis [47]. Moreover, adipogenic differentiation was impaired in 3T3-L1 preadipocytes containing hyperactivated MEK1 or constitutively expressing active ERK1/2 [47]. Consequently, the contradiction may be that ERK1/2 is necessary for initiating the differentiation process of preadipocytes, and afterwards, this signal pathway needs to be shut off for adipocyte maturation to continue [25]. Based on this line of thought, interventions in the ERK1/2 signalling pathway at different stages of the differentiation process have different effects on adipogenic differentiation even within the same cell type. On the other hand, various stimuli inversely regulate ERK1/2 phosphorylation in different cell types, which leads to contradictory effects on adipogenic differentiation. Phloretin promoted adipogenesis in mouse marrow stromal ST2 cells by inhibiting ERK1/2 [48]. Erythropoietin inhibited the adipogenic commitment of human BMSCs (hBMSCs) in vitro by increasing ERK1/2 phosphorylation level [49]. hAMSC adipogenic differentiation was inhibited by maintaining continuous ERK1/2 phosphorylation with FGF2 at a concentration of >10 ng/mL [50]. These results suggest that the role of the MEK–ERK1/2 signalling pathway on adipocyte differentiation depends on the intervention period, type of stimulation and cell type involved. Our results show that blocking ERK1/2 phosphorylation from the initial stage of induction suppresses hAMSC adipogenic differentiation in a concentration-dependent manner. Then, we verified whether SPRY4 regulates hAMSC adipogenic differentiation through the MEK–ERK1/2 signalling pathway. As shown in Figure 6, the enhancement of adipogenesis by SPRY4 was almost abolished by U0126, thereby confirming our hypothesis. Certainly, in order to find more precise targets for obesity treatment, further studies are required to elucidate the specific mechanism by which SPRY4 activates the ERK1/2 signalling pathway. Moreover, researches showed that white mature adipocytes are plastic cells able to reversibly transdifferentiate towards fibroblast-like cells maintaining stem cell gene signatures under appropriate paracrine or endocrine stimuli; this could be important to curb adipogenesis and the number of adipocytes [51]. Thus, further studies are needed to find out whether SPRY4 is involved in adipocytes transdifferentiate towards stem cells.

## Conclusion

We have elucidated the role of the SPRY4–ERK1/2 interaction in hAMSC adipogenic differentiation. We demonstrated that SPRY4 is positively associated with hAMSC adipogenic differentiation. Functional analyses revealed that of SPRY4 promotes hAMSC adipogenic differentiation in vitro and enhances ectopic fat formation in vivo effectively. Importantly, the molecular mechanism studies show that inhibiting the MEK–ERK1/2 pathway inhibits hAMSC adipogenic differentiation. SPRY4 increased ERK1/2 phosphorylation and activated the MEK–ERK1/2 pathway, thereby regulating the expression of genes involved in hAMSC adipogenesis. Our findings reveal the contribution of SPRY4 to hAMSC adipogenesis, and further identification of the cell type-specific signalling pathways in hAMSC adipogenesis, will help to expand the treatment options for combating obesity and the related health complications.

## Supplementary Material

Supplemental Material

## Data Availability

The datasets used and/or analysed during the current study are available from the corresponding author on reasonable request.
