# Supplementary material for: SPRY4 promotes adipogenic differentiation of human mesenchymal stem cells through the MEK–ERK1/2 signaling pathway
Source: Adipocyte. 2022 Sep 15;11(1):588–600. doi: 10.1080/21623945.2022.2123097 (PMC9481072; doi:10.1080/21623945.2022.2123097)
Supplement: Supplemental Material [file KADI_A_2123097_SM6675.zip › supplementary/Supplementary Figure legend and table.docx]

**Supplementary Figure 1. Characterization and identification of hAMSCs.**

(A) hAMSC morphology was assessed under a light microscope. (B) Adipogenic differentiation was assessed by oil red O staining on day 10. (C) Osteogenic differentiation was assessed by ALP staining on day 6. (D) Osteogenic differentiation was assessed by alizarin red S staining on day 12. (E) hAMSC phenotypes were identified by flow cytometry (n = 3 independent experiments with three biological repetitive tests, scale bar = 100 μm).

**Supplementary Figure 2. SPRY4 activated the MEK1–ERK1/2 signaling pathway.**

(A) Proteins were collected after knockdown of SPRY4 in hAMSCs. Then, SPRY4, p-ERK and T-ERK were detected with western blotting, and quantitative analysis of the intensity of protein expression in the indicated groups. (B) Under the same treatments except lentiviral SPRY4 overexpression in hAMSCs, SPRY4, p-ERK and T- ERK were detected with western blotting, and quantitative analysis of the intensity of protein expression in the indicated groups. GAPDH was used as the control for normalization (n = 3 independent experiments with three biological repetitive tests).

**Supplementary Table 1. All primers and siRNAs used in this study.**

| **Gene** | **Primer sequence** |
| --- | --- |
| SPRY4 | F: 5’-CTGACCAACGGCTCTTAGAC-3’ |
|  | R: 5’-GATGCACACTCCTTGCATTTAC-3’ |
| PPARG | F: 5’-CCTATTGACCCAGAAAGCGATT-3’ |
|  | R: 5’-CATTACGGAGAGATCCACGGA-3’ |
| C/EBPA | F: 5’-AGGAACACGAAGCACGATCAG-3’ |
|  | R: 5’-CGCACATTCACATTGCACAA-3’ |
| FABP4 | F: 5’-AGCACCATAACCTTAGATGGGG-3’ |
|  | R: 5’-CGTGGAAGTGACGCCTTTCA-3’ |
| LPL | F: 5’-ACAAGAGAGAACCAGACTCCAA-3’ |
|  | R: 5’-AGGGTAGTTAAACTCCTCCTCC-3’ |
| GAPDH | F: 5’-GGTCACCAGGGCTGCTTTTA-3’ |
|  | R: 5’-GGATCTCGCTCCTGGAAGATG-3’ |
| NC | r(UUCUCCGAACGUGUCACGU)dTdT |
| siSPRY4-1 | r(GACCAGCCAUGUGGAGAAU)dTdT |
| siSPRY4-2 | r(UCAACUAUGGCACGUGCAU)dTdT |
