# Supplementary figures and images for: SPRY4 promotes adipogenic differentiation of human mesenchymal stem cells through the MEK–ERK1/2 signaling pathway
Source: Adipocyte. 2022 Sep 15;11(1):588–600. doi: 10.1080/21623945.2022.2123097 (PMC9481072; doi:10.1080/21623945.2022.2123097)

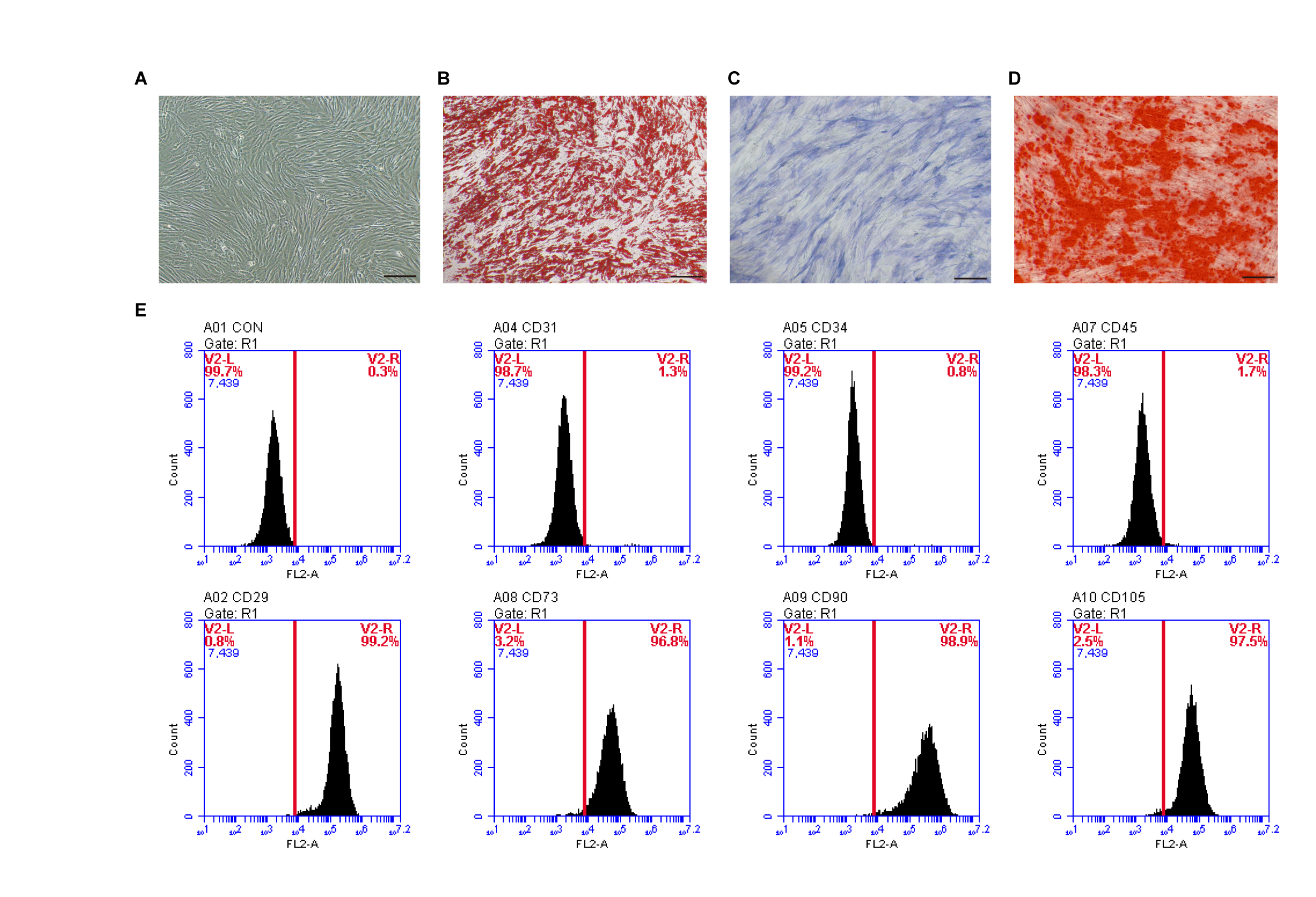

Supplement: Supplemental Material [file KADI_A_2123097_SM6675.zip › supplementary/FIG s1.tif]

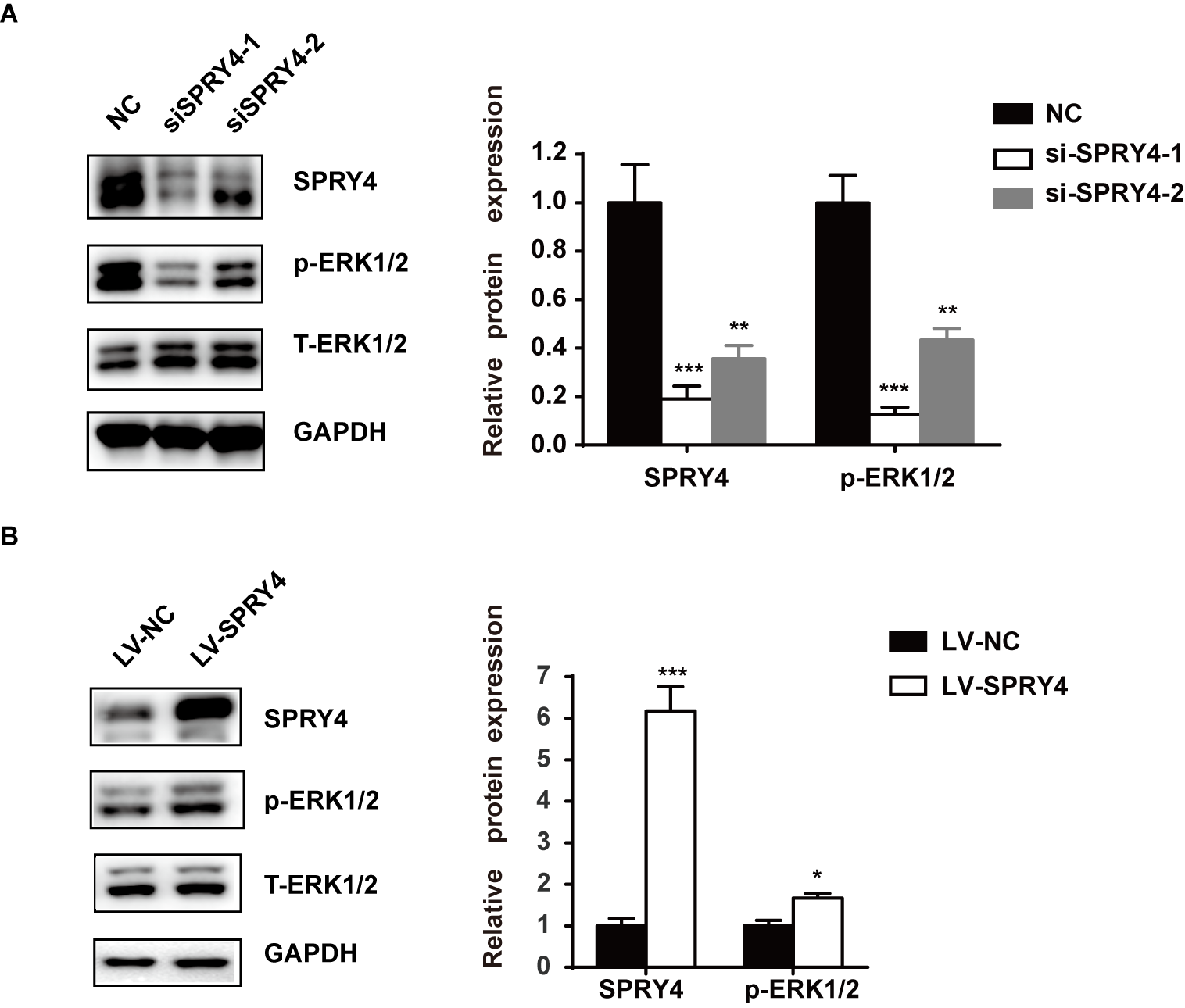

Supplement: Supplemental Material [file KADI_A_2123097_SM6675.zip › supplementary/FIG s2.tif]
